# Modified cranial closing wedge ostectomy in 169 stifles: considerations on potential factors associated with construct failures

**DOI:** 10.1111/jsap.70108

**Published:** 2026-02-25

**Authors:** J. Winter, C. Banks, G. Jones, R. Meeson

**Affiliations:** ^1^ Department Clinical Science & Services, Royal Veterinary College, The Queen Mother Hospital for Animals University of London Hatfield UK

## Abstract

**Objectives:**

The purpose of this study was to evaluate risk factors for construct failure in dogs undergoing unilateral modified cranial closing wedge ostectomy for cranial cruciate ligament disease.

**Materials and Methods:**

Hospital and radiographic data were collected retrospectively. A sub‐group for construct failure analysis was formed consisting of construct failure stifles and weight and plate matched control stifles. The following factors were assessed in relation to construct failure: plate size, plate position, proximal tibial flare, presence of adjunctive fixation and pre‐operative and post‐operative tibial plateau angle.

**Results:**

One hundred and sixty‐nine stifles were included within the overall data set, within which there was a major complication rate of 12% and a re‐operation rate of 8%. There were 10 construct failure stifles and 52 weight and plate matched controls in the construct failure analysis. We found no difference between construct failure and weight and plate matched controls for plate position, proximal tibial flare, presence of adjunctive fixation and pre‐operative or post‐operative tibial plateau angle. Visual assessment of plate size relative to weight showed that consistently larger plates were used in comparison to those recommended for tibial plateau levelling osteotomy.

**Clinical Significance:**

The lack of significance in any variable between construct failures and weight and plate matched controls is likely a type II error. Despite this, when planning a cranial closing wedge ostectomy consideration should be given to plate size, plate position, shape of the proximal tibia and the use of adjunctive fixation.

## INTRODUCTION

Cranial cruciate ligament disease (CCLD) is the most common cause of hindlimb lameness in dogs (Johnson et al., 1994) and results in functional stifle instability that manifests as cranial tibial thrust in locomotion (Slocum & Devine, 1983). The cranial closing wedge ostectomy (CCWO) was designed to reduce the tibial plateau angle (TPA) to 5°, eliminating cranial tibial thrust and dynamically stabilising the stifle (Slocum & Devine, 1984). However, there are undesirable consequences of the CCWO including tibial shortening, patella baja and tibial long‐axis shift (TLAS), which influence the resultant post‐operative TPA (Banks et al., 2023; Miles & Nielsen, 2023). The tibial plateau levelling osteotomy (TPLO) was subsequently developed as an alternative procedure (Slocum & Slocum, 1993) which would avoid these issues and has largely superseded other techniques (von Pfeil et al., 2018).

Despite its potential drawbacks, the CCWO is more readily accessible as it does not require a range of set radii crescentic saws and is considered by some surgeons to confer certain advantages. These include the elaboration of a larger proximal fragment which is useful in very small tibiae or in dogs with a disproportionately narrow proximal tibia. Likewise, it may arguably confer advantages to dogs with an excessive TPA where such an extreme TPLO osteotomy rotation would be required, leaving a long, thin tibial crest vulnerable to fracture. To maintain the CCWO advantages and yet minimise the impact of TLAS and improve the accuracy of TPA correction, the CCWO has undergone various modifications to the wedge angle and position (Christ et al., 2018; Frederick & Cross, 2017; Kovacs et al., 2023; Oxley et al., 2013; Terreros & Daye, 2020; Wallace et al., 2011).

Clinical studies of the CCWO have demonstrated good to excellent clinical outcomes as assessed by owners and veterinary surgeons (Campbell et al., 2016; Fontalba‐Navas et al., 2023; Frederick & Cross, 2017; Oxley et al., 2013). In direct comparison, the TPLO and CCWO appeared comparable (Corr & Brown, 2007; Oxley et al., 2013). Complication rates for CCWO have varied between 6.7% and 53%, with re‐operation rates between 1.8% and 35%. The predominant complications reported included late meniscal injury (0% to 11.8%), infection (5% to 23.5%), seroma (0% to 12.3%), implant failure (0% to 13.6%) and fracture of the tibia or fibula (0% to 17.6%) (Campbell et al., 2016; Corr & Brown, 2007; Fontalba‐Navas et al., 2023; Kovacs et al., 2023; Kuan et al., 2009; Oxley et al., 2013). There is significant variability between reports in terms of wedge apex, orientation and location of proximal and distal osteotomies and implant selection; however, the two reports documenting the isosceles CCWO technique stabilised with a locking TPLO plate describe relatively low complication rates (6% and 20%) and re‐operation rates (3.3% and 5.4%) (Fontalba‐Navas et al., 2023; Oxley et al., 2013).

One of the most problematic complications in all tibial osteotomies is failure of the bone–implant interface. In its mildest form, failure may lead to a variable return to pre‐operative TPA (“rock back”) with its resultant re‐generation of tibial thrust, through to complete construct failure (CF) rendering the limb mechanically incompetent. The aim of this study was to review overall complications with the isosceles CCWO in a large group of dogs and then to focus on factors relating to implant selection and implant positioning which may influence CF.

Based on clinical observations and biomechanical considerations, it was hypothesised that the following factors may influence CF: (1) plate size relative to body weight, (2) plate positioning within the proximal fragment, (3) tibial conformation and (4) the addition of a cranially positioned implant.

## MATERIALS AND METHODS

### Inclusion criteria and data collection

Clinical records were searched for dogs which had undergone an isosceles CCWO between February 2016 and December 2022, with follow‐up including radiographs to at least 6 weeks post‐surgery. Dogs that subsequently re‐presented for the contralateral stifle were included as separate joints for analysis.

Data on signalment, clinical examination findings, surgical management and follow‐up examinations were recorded. Cases were categorised by body weight and by TPLO plate (Depuy Synthes, Solothurn) size (2.0, 2.4, 2.7, 3.5 mm small, 3.5 mm standard and 3.5 mm broad).

All complications were classified according to Cook et al. (2010). Radiographs were analysed in medical image viewing software (Horos [Horos Project; Nimble]). TPA was measured on all pre‐operative, immediate post‐operative and follow‐up radiographs in the standard manner (Slocum & Slocum, 1993). Follow‐up radiographs were assessed for healing according to a previously published scheme (Oxley et al., 2013).

### Assessment of potential risk factors for bone–implant failure

Dogs with visible CF were separately analysed and compared to a sample group of weight and plate size matched control (WPMC) dogs without evidence of bone–implant failure. CF was defined as any change in implant or bone position or visible fractures identified on follow‐up radiographs. For each stifle joint included in the CF group, five stifle joints with the nearest body weight from the overall population for each plate size were selected without prejudice for the WPMC group. Where two dogs were of the same body weight, both were included.

The CF and WPMC groups were compared to assess the following a priori factors:

#### Implants additional to the TPLO plate

A descriptive assessment of additional implants placed in CF cases was made and then compared to WPMC.

#### Proximal tibial flare

**FIG 1:**
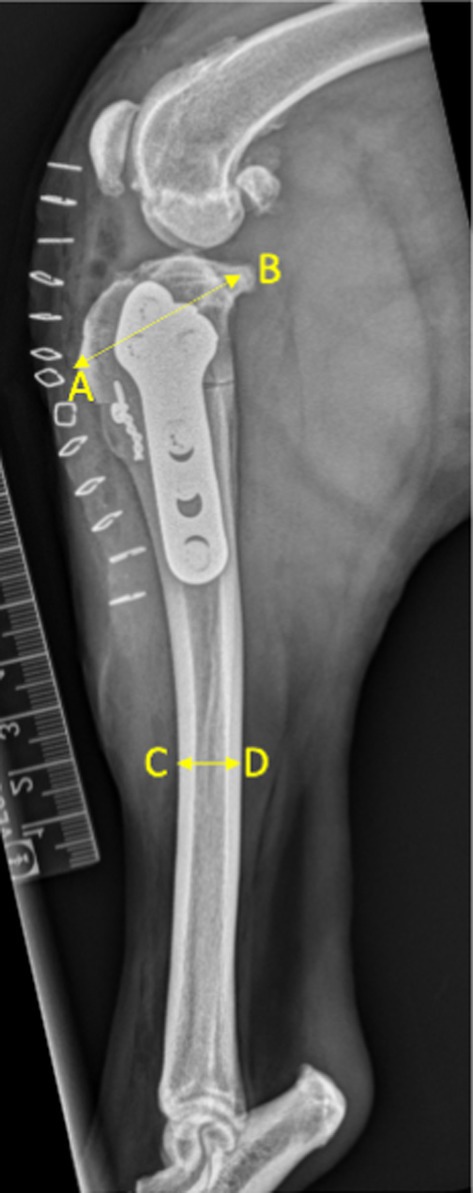
A mediolateral radiograph of the tibia of a CCWO demonstrating the measurements used to calculate proximal tibial flare. Length AB: proximal tibial depth and length CD: Isthmus of the tibia. CCWO, cranial closing wedge ostectomy.

From scaled mediolateral view radiographs, proximal tibial flare was calculated as follows (see Fig 1):

- Length AB – proximal tibial depth (distance from most proximal point of margo‐cranialis tibiae to the caudal aspect of the tibial plateau).
- Length CD – tibial isthmus.

Proximal tibial flare was calculated by expressing the ratio between proximal tibial depth and the tibial isthmus (AB/CD).

#### Craniocaudal plate position within the proximal fragment

**FIG 2:**
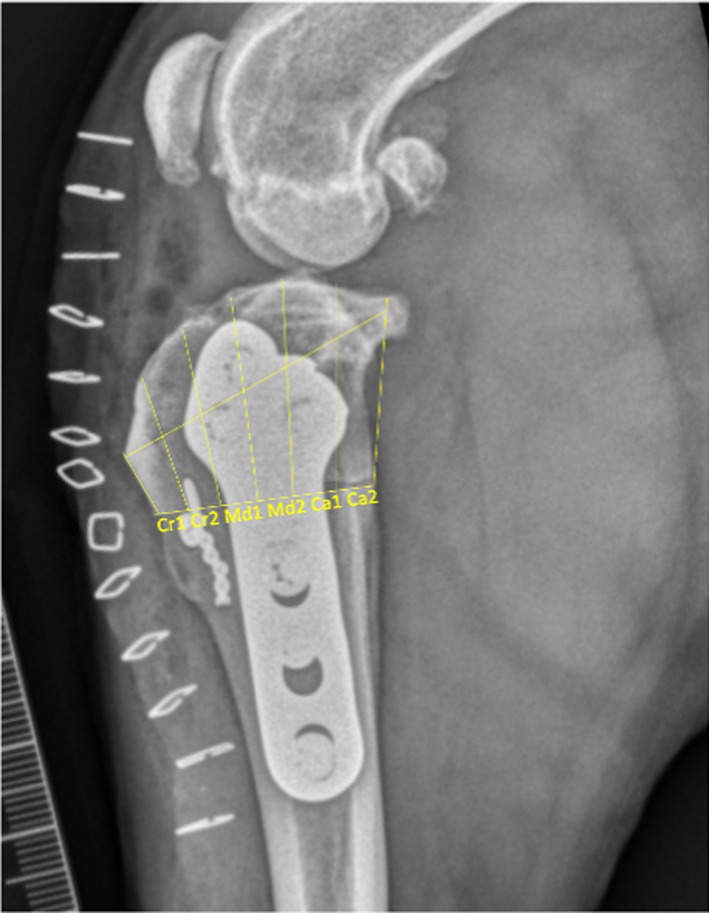
A mediolateral radiograph of the stifle of a CCWO demonstrating the assessment of plate position. The proximal fragment was divided into six segments (cranial 1 and 2, middle 1 and 2 and caudal 1 and 2). The screws in this plate sit within segments middle 1, middle 2 and caudal 1. This plate is therefore classed as caudal. CCWO, cranial closing wedge ostectomy.

To determine the cranial to caudal plate position, the proximal tibial section was divided into six equal‐sized sectors. The line AB and the length of the osteotomy were divided into six equal measurements. The points along each line were then connected, generating six sectors labelled cranial 1 and 2, middle 1 and 2 and caudal 1 and 2 (Fig 2). Plate screws were then recorded by which sectors they occupied. Plates were classed as cranial if they occupied a cranial sector and not a caudal sector. Plates were classified as caudal if they occupied a caudal sector but not a cranial sector. Plates were classified as central if the screw distribution was symmetrical.

#### Proximodistal plate positioning

The proximal screw closest to the osteotomy was selected, and the distance was measured between the screw head and the osteotomy. According to AO guidelines, each measurement was categorised as “acceptable” if the measurement exceeded the diameter of the screw head, or “unacceptable” if the measurement was smaller than the diameter of the screw head (Ruedi et al., 2007).

The CF group was compared to the remainder of the entire study population to assess body weight relative to plate size. This was compared using a visual assessment by plotting each dog's body weight against plate size for the CF and groups.

### Statistical analysis

Statistical analysis was performed in SPSS (Version 28.0.0.0, IBM). Descriptive statistics were used to describe the groups. Comparison between CFs and matched controls (WPMCs) were made after assessing for normality by the Shapiro test, and normally distributed data were analysed with an unpaired Student's *t*‐test. Ordinal data were analysed with a Mann–Whitney *U* test. Categorical data were analysed with a Fisher's exact test. Statistical significance was set at *P*<.05.

## RESULTS

### Study population

A total of 218 stifles (178 dogs) were identified of which 169 stifles from 135 dogs met the inclusion criteria. Affected breeds included crossbreed (68), West Highland White terrier (15), Staffordshire Bull terrier (15), Cockapoo (9), Cocker spaniel (8), Bulldog (5) and others (49). Dogs were female neutered (64), female entire (22), male neutered (65) and male entire (18). Mean age at time of surgery was 81 (±32) months. Median weight was 13.3 (range 4.1 to 46.3) kg. The left stifle was affected in 85 and the right stifle in 84. No significant co‐morbidities were seen in the population assessed.

All stifles were explored via mini medial arthrotomy. Complete CCL rupture was evident in 110/169 stifles, partial rupture was noted in 55/169, and no data were available for 4/169. Meniscal injury was present in 57/169 stifles of which all had either a caudal pole hemi‐meniscectomy or a partial meniscectomy at the surgeon's discretion. All CCWO were stabilised with a locking TPLO plate (Synthes) with plate sizes as follows: 2.4 mm (28), 2.7 mm (73), 3.5 mm small (8), 3.5 mm standard (42) and 3.5 mm broad (18). An additional implant was present in 99/169 stifles: cranial interfragmentary wire (IFW) with 0.8 mm orthopaedic wire (24), IFW with 1 mm orthopaedic wire (31), IFW with 1.2 mm orthopaedic wire (31), cranial pin and tension band wire (8), k‐wire alone (3), 2.0 mm locking compression plate (1) and one stifle had an extracapsular suture placed due to profound cranial tibial translation. Median wedge size taken was 9 mm (range 5 to 22).

Mean pre‐operative TPA was 29° (±6°) and immediate post‐operative TPA was 7° (±4°). Mean surgery time was 112 (±29) min and mean anaesthetic time was 212 (±45) min. All patients received peri‐operative intravenous cefuroxime at 20 mg/kg every 90 min. Fourteen intra‐operative complications were encountered in 13 stifles: cranial tibial artery haemorrhage (4), screw replacement due to inadequate screw length (4), pull‐through of the inter‐fragmentary wire (2), medial displacement of patella identified on post‐operative radiographs with capsular closure revised (1), broken wire (1), screw stripping (1) and a large transcortical gap requiring an additional plate (1).

All owners were instructed to crate rest their dogs for 6 to 8 weeks with short lead walks only. All dogs were discharged with a course of oral non‐steroidal anti‐inflammatories unless contraindicated. A one‐week course of post‐operative antibiotics was administered in 26 stifles: cephalexin 20 mg/kg orally twice daily (16), co‐amoxiclav 20 mg/kg orally twice daily (9), pradofloxacin 3 mg/kg orally once daily (1). Routine follow‐up was scheduled for 8 weeks from the date of surgery.

### Follow‐up and complications

First follow‐up was at a median of 57 days (range 8 to 1246). Radiographs were available in all cases. Visual lameness assessment (by a board certified surgeon) was a median of 0 (range 0 to 5) out of 5. Radiographic healing was complete in 45 (27%), good in 102 (60%) and poor in 22 (13%) stifles. Follow‐up TPA was a mean of 8° (±6°).

**FIG 3:**
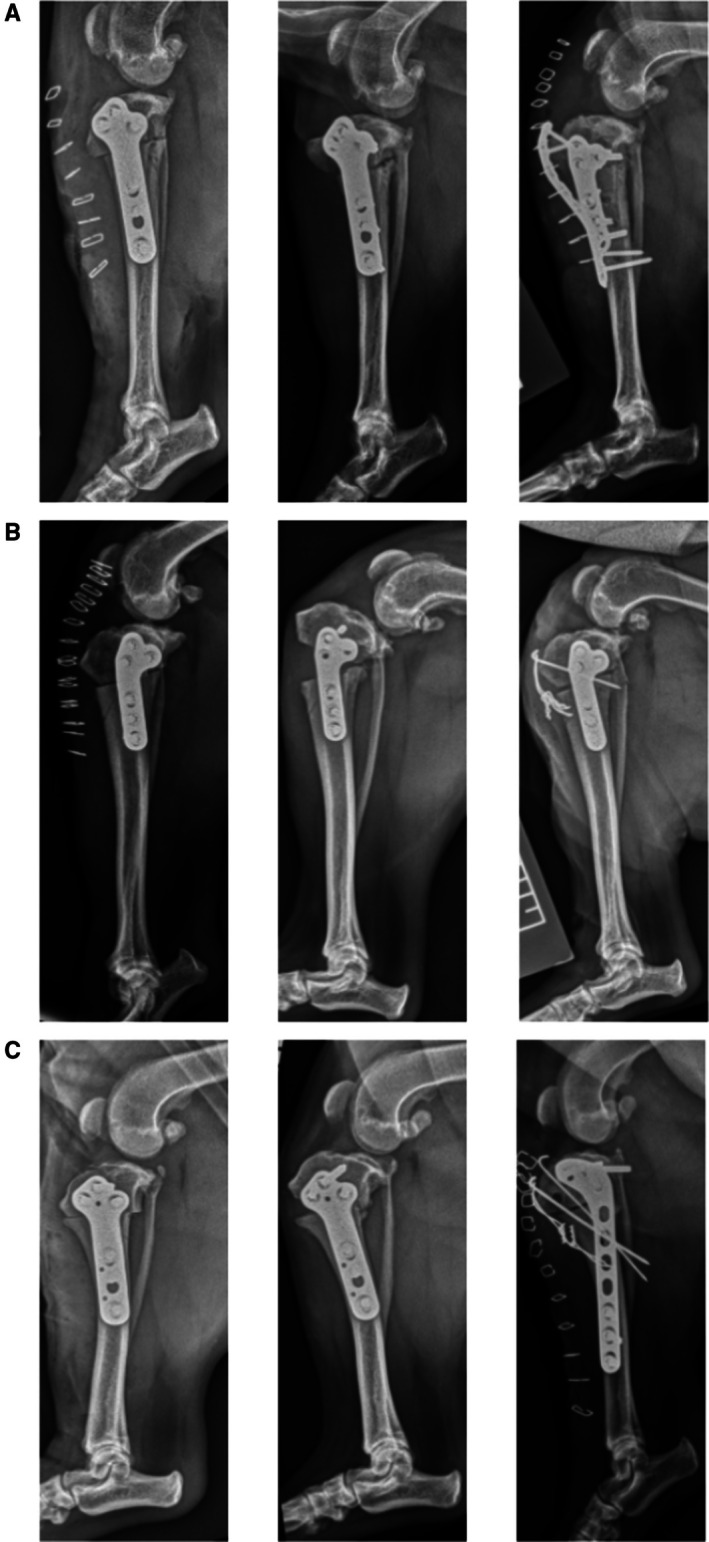
Radiographs taken from three cases in the construct failure group. Radiographs shown were taken immediately post‐CCWO, post‐failure and post‐revision surgery. (A) A case of failure by fracture of the proximal segment, (B) a case of failure by screw breakage and (C) a case of proximal segment instability due to bone failure and screws “cheese‐wiring” through the bone. CCWO, cranial closing wedge ostectomy.

Complications occurred in 29/169 stifles (17%). There were no catastrophic complications. Twenty‐two major complications were seen in 21 stifles (12%); eight had late meniscal injury (5%), four (2%) had surgical site infections diagnosed based on clinical signs (2) or by bacterial culture (2), and 10 had CFs within the peri‐operative period: Six had fracture of the proximal segment at the cranial aspect (Fig 3A); two had implant failure through screw breakage (Fig 3B), and two had proximal segment instability due to the implant pulling through “cheese‐wiring” the bone (Fig 3C). Of the 10 failures, 5/10 presented early with acute deterioration and were significantly more lame than expected, 4/10 presented at the time of routine re‐examination without severe lameness, and 1/10 presented at routine re‐examination with severe lameness and a history of having deteriorated 4 weeks prior to re‐presenting. Fourteen stifles underwent further surgery resulting in a re‐operation rate of 8%; eight late meniscal injuries were diagnosed and treated by mini medial arthrotomy and debridement of the lesion; two tibial tuberosity fractures were managed with surgical revision (four were conservatively managed); the two cases of proximal segment instability due to “cheese‐wire” bone failure were revised surgically in both stifles. Screw fracture implant failure was managed by revision surgery on one and conservatively in the other; and one implant infection underwent implant removal (remaining three managed with medical therapy only). Minor complications were seen in a total of 9/169 stifles (5%) and included acute lameness which resolved spontaneously (7), seroma (1) and restricted range of motion (1).

### Bone–implant failure analysis

Ten CF stifles (Table 1) were compared with 52 dogs in the WPMC group. There was no difference in median weight (*P*=.949) between groups. There was no difference in mean pre‐operative TPA (*P*=.772) which was 29° (±5) in the CF group and 29° (±5) in the WPMC group. There was no difference in mean post‐operative TPA (*P*=.195) which was 6° (±2°) in the CF group and 8° (±4°) in the WPMC group.

**Table 1:** Descriptive statistics for CF group

Signalment	Plate (synthes locking TPLO)	Additional fixation	Pre‐op TPA degrees	Post‐op TPA degree	Plate position	Proximal tibial flare	Complication	Management of complication
West Highland White terrier 10 years MN, 12 kg (BCS 7/9)	2.7 mm	None	29	5	Cranial	3.2	Bone fracture	2.0 mm LCP cranial and 2.4 mm TPLO plate caudal
Bulldog 4 years 2 months FE, 19 kg (BCS 5.5/9)	3.5 mm	None	30	5	Middle	3.1	Bone fracture	Conservatively managed
Cocker spaniel 5 years 11 months FN, 15 kg (BCS 6/9)	3.5 mm small	1.0 mm IFW	22	4	Caudal	3.5	Bone fracture	2.7 mm DCP supracondylar plate and 2.0 mm DCP supracondylar plate
English springer spaniel 9 years ME, 23 kg	3.5 mm	1.2 mm IFW	26	7	Cranial	2.7	Bone fracture, LMI	Conservative management, meniscal debridement
Mastiff 1 year 5 months MN, 43 kg (BCS 4/9)	3.5 mm	1.2 mm IFW	31	15	Middle	2.9	Bone fracture	Conservative management
West Highland White terrier 6 years 4 months FE, 11 kg	2.7 mm	1.0 mm IFW	34	6	Cranial	3.0	Bone fracture	Conservative management
Labrador 6 years 2 months FN, 26 kg (BCS 7/9)	3.5 mm	IFW pulled through and removed	28	5	Cranial	2.8	Bone failure	2.0 mm DCP supracondylar plate (inverted), 2.7 mm lag screw and 3.5 mm TPLO plate repositioned
Pembrokeshire Welsh Corgi – X, 1 years 11 months FN, 14 kg (BCS 8/9)	2.7 mm	None	25	9	Cranial	3.2	Bone failure	2.4 mm DCP supracondylar plate (inverted) and pin and cranial tension band (2 × 1.0 mm k‐wire, 0.8 mm orthopaedic wire)
Jack Russell terrier – X 6 years 2 months MN, 10 kg (BCS 5/9)	2.4 mm	None	37	8	Caudal	3.4	Implant failure – proximal screws broke	2.7 mm screws placed in proximal holes of 2.4 mm TPLO plate, cranial pin and tension bend (1.25 mm k‐wire, 0.8 mm orthopaedic wire)
Shih‐tzu – X 6 years 5 months FN, 8 kg (BCS 6/9)	2.4 mm	None	27	2	Caudal	4.0	Implant failure – screw loosening	Conservative management

Proximal tibial flare (larger number = wider proximal flare)

DCP Dynamic compression plate, cross breeds denoted by “‐X”, IFW Interfragmentary wire; LCP Locking compression plate

Additional cranial fixation was used in 4/10 of the CF group (four interfragmentary wires) and 33/52 of the WPMC group (29 interfragmentary wires, three pin and tension band wires and one k‐wire). There was no difference in failure rate with the addition of a cranial point of fixation (*P*=.718). Proximal tibial flare was not different between groups (*P*=.483) with a mean ratio of 3.2 (±0.4) in the CF group and 3.1 (±0.2) in the WPMC group. There was no difference in craniocaudal plate position between groups (*P* = .904), although a trend for more being caudally positioned in the WPMC group was seen: CF group, plates were cranial in 5/10, central in 2/10 and caudal in 3/10. In the WPMC group, plates were cranial in 21/52, central in 11/52 and caudal in 20/52. No statistical testing was performed for proximo‐distal plate positioning, as most plates were deemed within acceptable distance of the osteotomy (CF group 9/10, WMPC group 51/52).

**FIG 4:**
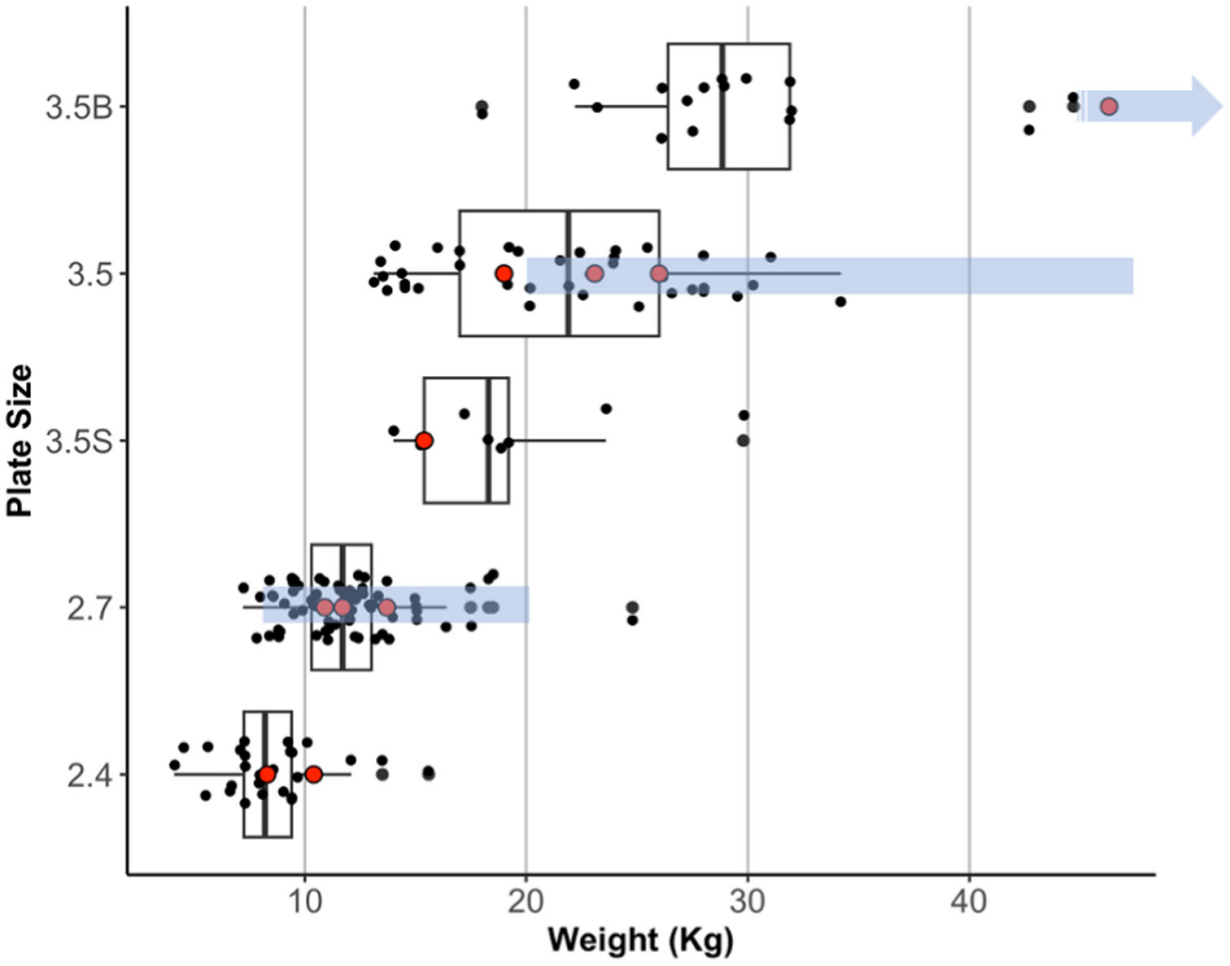
Plate size plotted against weight. Failures are represented by red dots, with all other cases represented by black dots. The blue bars represent the Synthes plate weight guidelines for TPLO. 3.5S = 3.5 mm small plate, 3.5B = 3.5 mm broad plate. TPLO, tibial plateau levelling osteotomy.

TPLO plate sizes in the CF group were 2.4 mm (2), 2.7 mm (3), 3.5 mm small (1), 3.5 mm standard (3) and 3.5 mm broad (1). TPLO plate sizes in the WPMC group were 2.4 mm (10), 2.7 mm (16), 3.5 mm small (5), 3.5 mm standard (16) and 3.5 mm broad (5). The relationship between plate size, weight and failure for the entire study population is plotted in Fig 4.

## DISCUSSION

The population of dogs presenting with cranial cruciate disease and managed with an isosceles CCWO is similar to other studies (Fontalba‐Navas et al., 2023; Oxley et al., 2013). Although the overall total complication rate and re‐operation rate for this group of 169 stifles was similar to Oxley et al. (74 CCWOs), the nature of the complications was different. The predominant reasons for re‐operation herein were late meniscal injury and CF, whereas there were no cases of CF in the Oxley publication.

It should be noted that the mechanical environment for the CCWO is different to that of the TPLO. The proximal fragment in a TPLO does not incorporate the insertion of the quadriceps muscle and has a high contact area of the two sides of a single compressed radial cut, providing good inherent stability. The CCWO, however, requires the surgeon to make two parallel freehand cuts and maintain their alignment. Should the surgeon fail to achieve parallel cuts, correct alignment cannot be maintained in the frontal plane without compromising load‐sharing between the two fragments. The CCWO proximal segment remains attached to the quadriceps mechanism; hence, it is considered that the proximal segment and the implant(s) stabilising it have higher biomechanical requirements than the equivalent TPLO. Dogs with a higher pre‐operative TPA will require a larger wedge ostectomy to bring the post‐operative TPA to the desired 5°. This could potentially distalise the most proximal point of the margo cranialis tibia and increase the working length of the quadriceps for the same amount of stifle flexion, which may increase the avulsion forces on the proximal segment (Shimada et al., 2023). Indeed, there were differences in the sample population with steeper TPAs in smaller dogs included in this study compared to Oxley et al. However, in comparisons between CF and WPMC, TPA did not influence failure rate in our study. Notably, 30 (18%) stifles had excessive pre‐operative TPA (>34°) but only one of these failed. A common theme relating to TPA was a mild under‐correction, with a mean post‐operative TPA of 7°. This is consistent with a recent *in silico* study which showed an inconsistency in achieving the target TPA with the isosceles CCWO method (Banks et al., 2023). While of interest, we consider this unlikely to be clinically relevant as post‐operative TPA up to 14° has been shown to adequately stabilise the CCLD stifle (Duerr et al., 2008).

As the biomechanical requirements of the CCWO are different to the TPLO and likely more demanding, related factors of implant selection and positioning were also considered potential risk factors for CF. It was hypothesised that in the dogs which had CF the plate size relative to their body weight may have been a contributing factor. One of the proposed reasons a CCWO may be chosen has been to establish a bigger proximal fragment to allow a bigger plate to be placed. In the vast majority of cases, dogs within certain weight groups were having a larger plate placed than the published Synthes recommended range for TPLO (Fig 4). Notably, where implants have failed (*n* = 2), both cases had relatively undersized plates compared to the majority of their cohorts although they were within the TPLO plate guidelines provided by Synthes. Furthermore, neither of these cases had an additional tension‐relieving device applied, such as a pin and tension band wire, which may have improved construct stability and reduced the load encountered by the plate. However, with such small group sizes, it remains a consideration. A further plate factor to consider was whether the selection of a bigger plate in relatively smaller bone stock may create weakness within the proximal tibia, predisposing the bone to fissuring and fracture. Visual review of our dataset does not obviously support this; however, there could be unforeseen interplay of application of adjunctive implants and the relative positioning of the larger plate within the proximal segment.

Oxley et al. included a cranial interfragmentary wire to facilitate reduction of the ostectomy and provide additional resistance to the pull of the quadriceps (Oxley et al., 2013), although this is not a true‐tension relieving device, such as a pin and figure‐of‐eight tension band wire. Fracture of the proximal fragment was noted in six stifles, four of which had an additional interfragmentary wire and one of which had an interfragmentary wire placed but it pulled through and was removed at the time of surgery. In these stifles, fracture lines propagated through the wire hole in the proximal fragment and then onto the cranial‐most screw hole. Consequently, it was considered that the interfragmentary wire hole may contribute to failure and it was no longer routinely implemented at this institution. No further fractures of the proximal fragment were noted after this change was implemented; however, two screw failures and one bone failure occurred without a cranial implant. In these three failures, the plate screws either broke, loosened or “cheese‐wired” through the bone. It is conceivable, but supposition, that a tension relieving additional implant may have mitigated these events.

Although there may be an apparent increase in available bone stock when performing a CCWO compared with the mid‐caudally positioned TPLO osteotomy, the proximal tibia is triangular in cross‐section, tapering to become unicortical at the most cranial extent of the cranialis tibialis fossa. A plate placed in the very cranial region of the osteotomy may be in bone with reduced holding capacity. Likewise, the application of a larger plate with larger screws creating larger holes could also weaken this thinner cranial bone stock further. The relationship of cranial to caudal plate position was semi‐quantitatively assessed in this study and found no statistical difference between groups. Nonetheless, the authors would still suggest it is advisable to position the plate in a more caudal and proximal position to effectively use the widest portion of the proximal tibia for screw purchase.

Plate positioning is also affected by the shape of the proximal tibia (*i.e*. the space within which to place the plate), and this was considered another potential factor, with some dogs having relatively wider proximal tibiae than others. Although a wider proximal tibia may give more room to place the same‐sized plate more caudally in improved bone stock, there may be unseen mechanical consequences. Considering the mechanism of failure, the CCWO creates a torque lever arm construct: the quadriceps avulsion tension from the patellar ligament is the line of action, which creates a moment arm which then wants to rotate against the plate screws, and ultimately the most caudally positioned screw acts as an axis. A proportionately wider proximal tibia and a greater distance between the caudal screw and the most proximal aspect of the margo cranialis tibiae results in a greater moment arm and potentially increases the likelihood that a CF will occur. The wide tibia scenario is seen commonly in chondrodystrophic dogs and smaller dogs with an excessive TPA and caudal bowing of the tibia. Indeed, three of four cases where the entire proximal fragment was destabilised (due to implant failure or bone failure) were chondrodystrophic dogs. Both cases of screw failure were cases with 2.4 mm TPLO plates positioned relatively caudally within the proximal fragment. Notably, the 2.4 mm synthes TPLO plates are shaped such that the proximal screws are positioned further caudally leading to an increased moment arm. There was no statistical difference in tibial flare within our study, which may have been due to the proposed balance of mechanical loading and bone quality cancelling each other out. Based on our experience, we suggest considering a cranial pin and tension band on a case‐by‐case basis when a larger lever arm effect is possible (*i.e*. caudally positioned plate on a wide proximal tibia).

The rate of late meniscal injury reported (5%) is comparable to other studies (Corr & Brown, 2007; Oxley et al., 2013). The rate of surgical site infection (2.4%) is also within the expected limits of elective veterinary orthopaedic surgery (Weese, 2008). Both late meniscal injury and surgical site infection can occur as late complications and so it is possible that they could be underreported given the lack of active long‐term follow‐up. Likewise, the minor complications reported are solely those that re‐presented to our hospital. Notably, antibiotics were prescribed at discharge in several cases, and due to the retrospective nature of the study, the rationale was unclear. Our institution promotes judicious use of antibiotics and does not support post‐operative antibiosis in elective orthopaedic cases.

This study has multiple limitations inherent to it being a retrospective study as discussed. The lack of long‐term follow‐up for all cases prevents comment on long‐term outcome, although the focus was to better understand CFs in CCWOs. While all CCWOs were planned according to the method described by Oxley et al., in many cases, the wedge removed was not an exact isosceles triangle which highlights the difficulty in achieving two parallel cuts and an isosceles wedge. The main limitation is the small sample number of failures that may have prohibited effective analysis of factors that predispose to complications. Either the lack of significance in any variable between CFs and WPMCs is a resulting type II error, or the CFs might have been related to factors not analysed in this study. Nonetheless, based on the cases studied herein, the authors find the following points useful to consider for the planning and the execution of the isosceles CCWO:

- Consider the cranio‐caudal and proximodistal positioning of the plate within the proximal fragment.
- Consider the location and impact of interfragmentary wire holes in the proximal fragment and the potential for these to create a stress riser.
- Consider that in wide proximal tibiae with caudally positioned implants, there could be a relatively large moment arm, and so a cranial implant may be worth considering.

Complication rates were similar to previous studies; however, the nature of the complications was different. We saw several CF which were not reported previously with this technique. We were unable to effectively identify any factors that predispose to failure; however, this is likely a type II error as a consequence of the low number of failures. Further biomechanical studies are required to better understand the impact of plate size and positioning, addition of a cranial implant and shape of the proximal tibia on the failure rate of CCWO. Until then, careful consideration should be given to the aforementioned variables when planning a CCWO.

## Author contributions

**J. Winter:** Conceptualization; investigation; writing – original draft; methodology; writing – review and editing; data curation; project administration; formal analysis. **C. Banks:** Investigation; data curation; writing – review and editing. **G. Jones:** Methodology; statistics; writing – review and editing. **R. Meeson:** Conceptualization; methodology; writing – review and editing.

## Conflict of interest

None of the authors of this article has a financial or personal relationship with other people or organisations that could inappropriately influence or bias the content of the paper.

## Data Availability

The data that support the findings of this study are available from the corresponding author upon reasonable request.
